# The role of mentors in addressing issues of work–life integration in an academic research environment

**DOI:** 10.1017/cts.2019.408

**Published:** 2019-10-15

**Authors:** Dennis R. Durbin, Stephanie C. House, Emma A. Meagher, Jenna Griebel Rogers

**Affiliations:** 1 Department of Pediatrics, The Ohio State University College of Medicine, Columbus, OH, USA; 2 The Abigail Wexner Research Institute, Nationwide Children’s Hospital, Columbus, OH, USA; 3 Institute for Clinical and Translational Research, University of Wisconsin-Madison, Madison, WI, USA; 4 Department of Medicine, Perelman School of Medicine, University of Pennsylvania, Philadelphia, PA, USA

**Keywords:** Mentoring, academic medical center, professional development, mentor training, work–life integration, work–life conflict

## Abstract

**Introduction:**

There is growing evidence for both the need to manage work–life conflict and the opportunity for mentors to advise their mentees on how to do this in an academic research environment.

**Methods:**

A multiphase approach was used to develop and implement an evidence-informed training module to help mentors guide their mentees in issues of work–life conflict. Analysis of existing data from a randomized controlled trial (RCT) of a mentor training curriculum (n = 283 mentor/mentee dyads) informed the development of a work–life mentoring module which was incorporated into an established research mentor training curriculum and evaluated by faculty at a single academic medical center.

**Results:**

Only 39% of mentors and 36% of mentees in the RCT indicated high satisfaction with the balance between their personal and professional lives. The majority (75%) of mentors and mentees were sharing personal information as part of the mentoring relationship which was significantly associated with mentees’ ratings of the balance between their personal and professional lives. The effectiveness of the work–life module was assessed by 60 faculty mentors participating in a mentor training program at an academic medical center from 2013 to 2017. Among the respondents to the post-training survey, 82.5% indicated they were very/somewhat comfortable addressing work–life issues with their mentees as a result of the training, with significant improvements (p = 0.001) in self-assessments of mentoring skill in this domain.

**Conclusions:**

Our findings indicate that a structured training approach can significantly improve mentors’ self-reported skills in addressing work–life issues with their mentees.

## Introduction

There is a growing body of evidence indicating that work–life integration is one of the most pressing challenges facing faculty in academic medical centers [1–7]. Work–life conflict has been identified as a key factor affecting faculty retention in academic medicine [5], particularly among women [4]. Most leading academic medical centers have developed and implemented institutional policies to address work–life challenges, including tenure deferment, part-time employment status, maternity/paternity leave, and job sharing [8,9] However, evidence suggests that there has been variable uptake and acceptance of these policies [1,3,9].

Complementing institutional policies to promote work–life integration, effective mentoring is a critical determinant for career satisfaction in academic medicine [2,10,11]. However, advising about balancing work and family life was the mentoring role least likely to be reported by junior faculty in a large national survey of K award recipients, reported by only about 20% of both male and female junior faculty with dedicated mentors [2]. Dissatisfaction with the balance between personal and professional life was also common among both men (40%) and women (52%) in this sample. Given the growing evidence base for both the need to manage work–life conflict and the opportunity for mentors to provide role modeling, support, and guidance to their mentees on how to do this in an academic research environment, a structured approach to addressing work–life issues in academic mentoring is needed [1,2,4,5].

*Total Leadership* ^®^ is a leadership development program created by Stewart Friedman at the University of Pennsylvania Wharton Business School [12]. It is unique among leadership development programs in that it defines successful leadership as a function of a person’s ability to identify and align specific goals in four domains of life: work, family, community, and self. Achieving and sustaining these so-called “four-way wins” are considered central to becoming a more effective leader. *Total Leadership* provides a structured series of activities, which includes designing behavior change experiments to produce sustainable progress toward achieving self-defined goals in the four life domains with the support of “coaching teams,” consisting of other program participants. *Total Leadership’s* structured approach made it an attractive program to adapt into a training curriculum to encourage mentors in an academic research setting to address issues of work–life integration with their mentees.

We approached this initiative with a three-phase longitudinal research program. In Phase 1, we sought to understand the extent to which academic research mentors and mentees share information from their personal lives as part of the mentoring relationship, and how this impacts mentees’ assessments of their satisfaction with the balance between their personal and professional lives. For this phase of the project, we utilized baseline data from a prior randomized controlled trial (RCT) of a mentor training program known as *Mentor Training for Clinical and Translational Researchers* [13,14]. In Phase 2, we utilized results from the Phase 1 analysis to adapt elements of *Total Leadership* into a work–life mentoring module and incorporated this into the established research mentor training curriculum. Finally, in Phase 3, we implemented and evaluated the self-reported effectiveness of the work–life mentoring module as assessed by faculty mentors at the University of Pennsylvania Perelman School of Medicine and its affiliate, the Children’s Hospital of Philadelphia, who were participating in the mentor training curriculum.

## Materials and Methods

### Approach for Phase 1

Analyses for Phase 1 drew on the baseline data from a RCT designed to test the effectiveness of a research mentor training curriculum, *Mentor Training for Clinical and Translational Researchers* [13,14]. Data were collected via structured interviews in 2010 from 16 academic health centers across the USA and Puerto Rico. The sample included 283 faculty research mentors and 283 of their matched mentees, who consisted of early career faculty, postdocs, and graduate students. All interviews were conducted in person by trained research assistants at each site. The original clinical trial data collection was approved by the University of Wisconsin-Madison IRB (IRB #: M-2010-1053). The current analysis was exempt from IRB review given that it involved the analysis of existing data recorded in a manner by which subjects could not be identified.

Baseline data collected from both mentors and mentees on a wide range of issues relevant to a mentoring relationship included the validated Mentoring Competency Assessment (MCA), in which mentors’ skills are rated on a 7-point Likert scale ranging from 1 (not at all skilled) to 7 (extremely skilled) [15]. Other assessments included satisfaction with the respondents’ professional life, as well as the balance between their personal and professional lives, also measured on a 7-point Likert scale ranging from 1 (very unsatisfied) to 7 (very satisfied), and an assessment of the climate of the mentee’s work environment, ranging from 1 (very negative) to 7 (very positive). Among the items characterizing the mentoring relationship was one asking respondents to rate the degree to which they know about each other’s personal life (e.g., family, hobbies, interests outside of work) on a 7-point scale ranging from 1 (We know nothing about each other’s personal life) to 7 (We know a lot about each other’s personal life). Data from the baseline surveys were used in the current analysis to avoid introducing any effect of the training program. Our primary outcome of interest was the *mentee* rating of work–life satisfaction. To facilitate interpretation of analyses, we collapsed the 7-point scale into a three-level outcome variable: low satisfaction (ratings of 1–3), moderate satisfaction (ratings of 4 or 5), or high satisfaction (ratings of 6 or 7).

Descriptive statistics were used to summarize the distribution of mentor and mentee ratings of professional satisfaction as well as work–life satisfaction. Bivariate analyses were then conducted on a number of candidate mentor, mentee, and mentoring relationship/environmental factors to explore their association with mentee’s assessments of high work–life satisfaction. Factors that were associated with mentee work–life satisfaction were entered into a multivariable logistic regression model to ascertain the independent contribution each factor had on mentee work–life satisfaction. Odds ratios and 95% CI were calculated.

### Approach for Phase 2

Phase 2 activities consisted of “off-line” development of a new module in the mentor training curriculum through the adaptation of *Total Leadership* content, informed by the analyses conducted in Phase 1. Existing content in the 8-hour *Mentor Training for Clinical and Translational Researchers* curriculum is organized into roughly 1-hour standalone modules covering a variety of topics (introduction, maintaining effective communication, aligning expectations, assessing understanding, addressing equity and inclusion, fostering independence, promoting professional development, and articulating a mentoring philosophy) [13]. The *Total Leadership*^®^ program consists of a longitudinal series of exercises that culminate in the design and implementation of a behavior change experiment intended to better align the goals one identifies in each of the four domains of life: work, family, community, and self [12]. We adapted and integrated selected content from the program into the structure of the mentor training curriculum, resulting in a new module entitled *Enhancing Work–Life Integration*, with a companion facilitator’s guide.

The first activity requires participants to define a personal vision statement to identify what is most important to them. A “four-way assessment” is then conducted so participants can identify discrepancies between their perceived relative importance of each life domain with how much time and attention they are currently spending in that domain. Informed by these activities, participants design a behavior change experiment, intended to mitigate a discrepancy between the perceived level of importance in each domain with one’s actual allocation of time and attention. Participants are divided into groups of 3–4 individuals to serve as “coaches” for one another, troubleshooting the implementation of experiments, and providing mutual support and accountability to ensure that experiments were completed. All work–life integration experiments are designed to be conducted over the 12-week time period during which the mentor training curriculum is implemented.

In-person activities are supplemented by readings from the *Total Leadership* book [12], which was provided at no cost to workshop participants. Materials developed for the work–life mentoring module are available via the CIMER website https://cimerproject.org/ along with materials for all other mentor training topics.

### Approach for Phase 3

The research mentor training program has been offered each year since 2012 to faculty in the Tenure, Research and Clinician-Educator tracks at the “late Assistant Professor” (i.e., years 7–9 of appointment) rank or higher at the University of Pennsylvania Perelman School of Medicine. Participation is voluntary and is limited to a maximum of 15 faculty per session. The *Enhancing Work–Life Integration* module was developed in 2012 and initially integrated into the curriculum in 2013, resulting in a mentor training curriculum of five 2-hour sessions spaced 2–3 weeks apart. Prior to the start of each program, participating faculty completed a baseline survey ascertaining demographic information, characteristics of their mentoring experience, and self-assessments of a number of mentoring skills and behaviors using the MCA [15]. Faculty were surveyed within 1 week following the completion of the training program in order to ascertain feedback on the content and implementation of the training program, as well as any self-reported changes in mentoring skills assessments. New survey items were developed specific to the *Enhancing Work–Life Integration* module and incorporated into the standard assessment of the mentor training curriculum. Implementations for years 2013–2015 used a pre–post survey design. For implementations in 2016 and 2017, a retrospective pre–post survey design was used. This adjustment was made to reduce respondent fatigue as the retrospective pre-post was found to be a reliable means of self-assessing skill gains. The questions remained the same as in previous years, but faculty were only asked to assess skill gains at the completion of the training. The data collection for this phase of work was approved by the IRB at the University of Wisconsin-Madison (protocol #s: 2017-0026, 2016-0458, 2015-0871, 2015-0042, 2013-0732).

Data from both baseline and post-training surveys were used to assess the implementation and effectiveness of the new work–life mentoring module. Descriptive statistics were used to summarize the distribution of faculty characteristics, their ratings of specific mentoring skills and behaviors, and their feedback on components of the *Enhancing Work–Life Integration* module. The Wilcoxon signed-rank test was used to compare faculty assessments of specific mentoring skills and behaviors after vs. before participating in the mentor training program. *P* values were calculated for the difference in median ratings pre- vs. post-training.

## Results

### Phase 1 Results

The majority of mentors in the RCT were male (60%), white (91%), and had a mean age of 50.5 years (range: 31–81 years). Most were full or associate professors and reported extensive mentoring experience (average of 15 years, standard deviation 8.0 years). The mentors’ most common research focus area was clinical research (66%) and the remainder included laboratory, behavioral, and community engaged research. The mentees’ mean age was 35.9 years (range: 25–61), 42% were male and 74% self-identified as white, with 30% self-selecting other racial categories. Most mentees were funded by career development awards or postdoctoral fellowships. The majority conducted clinical research (69%) and the remainder were engaged in the full spectrum of clinical and translational research (for more information, see Pfund *et al*. 2014).

**Fig. 1.**
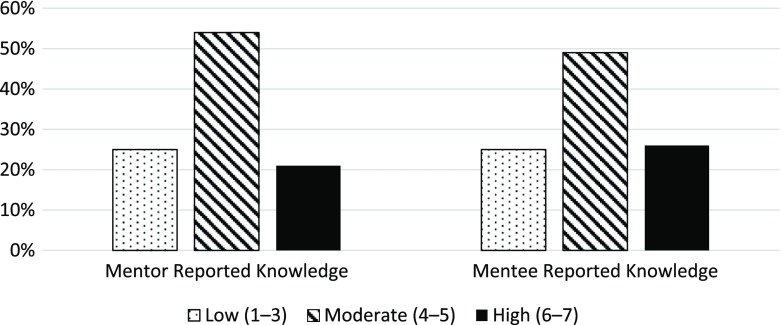
Phase 1 results – distribution of respondents’ ratings of knowledge of each other’s personal lives for both mentors and mentees.

Mentors and mentees had a similar distribution (weighted kappa = 0.33, 95% CI 0.24, 0.42) of responses to the question about knowledge of each other’s personal lives. Fig. 1 provides the distribution of responses for both mentors and mentees, indicating that approximately 3 out of 4 respondents in each group indicated moderate to high knowledge of each other’s personal lives.

**Fig. 2.**
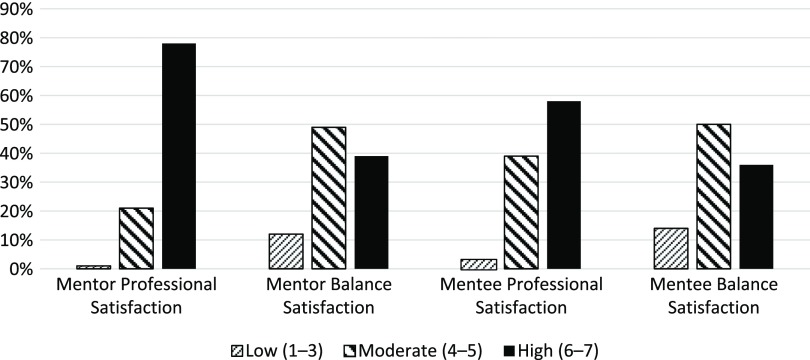
Phase 1 results –distribution of satisfaction with professional life, as well as the balance between personal and professional lives for both mentors and mentees.

Fig. 2 provides the distributions of the ratings of satisfaction with professional lives, as well as the *balance* between personal and professional lives, for both mentors and mentees. The majority of both mentors and mentees indicated fairly high ratings of satisfaction with their professional lives, with 78% of mentors and 58% of mentees indicating high satisfaction for this domain. The distribution of ratings for the *balance* between personal and professional lives indicated lower ratings of satisfaction, with only 39% of mentors and 36% of mentees indicating high satisfaction. Although the overall distributions of ratings for personal/professional life satisfaction are similar for mentors and mentees, they were not significantly correlated with one another (weighted kappa = 0.02).

Because the mentee’s rating of satisfaction with the balance between their personal and professional life was our primary outcome of interest, we examined the association between several mentor, mentee, and mentoring relationship characteristics with the mentee’s rating, grouped as: high (6, 7) vs. moderate/low (1–5) satisfaction. There was no association (*p* > 0.05) between the mentee’s satisfaction rating and the mentor’s or mentee’s gender or race, the mentor’s age or years of mentoring experience, the mentee’s academic rank or productivity as measured by number of grants submitted, or indicators of specific characteristics of the mentoring relationship such as responsiveness of mentors and helpfulness of feedback as assessed by mentees. Notably, there was no evidence (*p* = 0.56) for an association between gender concordance in the mentoring relationship and the mentee’s satisfaction with the balance between their personal and professional lives. In addition, there was no association between the mentee’s satisfaction with the balance between their personal and professional life and their own assessment of the knowledge of each other’s personal lives (*p* = 0.07).

Conversely, an association was noted (*p* < 0.05) between the mentee’s personal/professional balance satisfaction and the mentor’s academic rank; there was a “U-shaped” relationship, with lower ratings of personal/professional satisfaction among mentees with associate professor mentors. Associations were also found with the mentee’s assessment of the work climate (*p* = 0.03), the mentee’s assessment of the overall quality of their mentoring (*p* = 0.03), the mentee’s age (younger age associated with higher satisfaction, *p* = 0.01), and finally, the *mentor’s* reported knowledge of each other’s personal life (*p* = 0.03).

Table 1 provides results of the multivariable logistic regression analyses. After adjustment for all variables in the model, only the academic rank of the mentor (i.e., Professor), the mentee’s (younger) age, and the mentor’s knowledge of each other’s personal lives remained significantly associated with high mentee satisfaction in the balance between their personal and professional lives. Mentees whose mentors reported a high degree of shared personal knowledge were over twice as likely to report high satisfaction with the balance between their personal and professional lives as compared to mentees whose mentors reported low or moderate knowledge of each other’s personal lives. This finding is notable in light of the distribution of the mentors’ satisfaction with their own personal/professional balance, suggesting that independent of their own personal/professional satisfaction, their knowledge of their mentee’s personal life is associated with improved perceptions of personal/professional satisfaction on the part of mentees.

**Table 1.** Phase 1 results – factors associated with Mentees’ assessments of satisfaction with the balance between their personal and professional lives (outcome is high satisfaction vs. low/moderate)

Variable name	Unadjusted Odds ratio (95% CI)	Adjusted Odds ratio (95% CI)
Mentor rank		
Assistant professor	2.47 (1.08, 5.64)	2.13 (0.9, 5.04)
Associate professor	Reference	Reference
Professor	1.84 (1.04, 3.25)	1.99 (1.09, 3.65)
Mentees’ rating of climate of work environment		
Low/Moderate (1–5)	Reference	Reference
High (6–7)	1.94 (1.09, 3.47)	1.62 (0.86, 3.05)
Mentees’ rating of overall mentoring quality		
Low/Moderate (1–5)	Reference	Reference
High (6–7)	1.89 (1.06, 3.35)	1.48 (0.79, 2.78)
Mentors’ rating of knowledge of each other’s personal life		
Low/Moderate (1–5)	Reference	Reference
High (6–7)	1.87 (1.05, 3.34)	2.18 (1.18, 4.03)
Mentees’ age	0.95 (0.91, 0.99)	0.94 (0.90, 0.98)

### Phase 3 Results

From 2013 to 2017, a total of 60 faculty participated in mentor training sessions which included *Enhancing Work–life Integration*. Participant characteristics (*n* = 55 completed responses) are summarized in Table 2. Participants provided mentoring to a wide range of research trainees including undergraduates, PhD and Masters students, medical students, postdoctoral fellows, and medical specialty fellows, as well as junior faculty.

**Table 2.** Phase 3 results – characteristics of the faculty participating in the Research Mentor Training program at the University of Pennsylvania Perelman School of Medicine, 2013–2017

Participant characteristic	Number (%) *n* = 55 respondents^a^
Sex	
Female	29 (52.7)
Male	21 (38.2)
Missing	5 (9.1)
Median age (Range), Years	45 (36–70)
Race	
White	37 (67.2)
Black	3 (5.5)
Asian	10 (18.2)
Other/Missing	5 (9.1)
Median years of mentoring experience(Range)	5 (1–25)
Academic title	
Assistant professor	25 (45.5)
Associate professor	17 (30.9)
Professor	6 (10.9)
Other	7 (12.7)
Primary type of research^b^	
Clinical	34 (61.8)
Behavioral	8 (14.5)
Basic (laboratory-based)	13 (23.6)
Translational	12 (21.8)
Other	10 (18.2)

a There were a total of 60 faculty participants, 55 (91.6%) of whom completed pre-training surveys.

b Respondents could indicate more than one type of research.

Feedback on the overall mentor training program was positive with 46/50 (92%) respondents indicating the training was a valuable use of their time and 44/50 (88%) indicating they were likely or very likely to recommend the training to a colleague. Further, 47/50 (94%) indicated that they had already made or were planning to make changes in their mentoring practice as a result of the training. Feedback on the *Enhancing Work–life Integration* module demonstrated that 33/40 (82.5%) respondents self-rated their behavior change experiment as moderately or very successful, and 45/47 (95.7%) respondents were possibly or very likely to continue the experiment following the training. Finally, among the 40 respondents to the post-training survey question, “How comfortable would you be addressing work–life integration with your mentees,” 15 (37.5%) indicated they were very comfortable, 18 (45%) indicated they were somewhat comfortable, 6 (15%) indicated that they were somewhat uncomfortable, and only 1 (2.5%) indicated he/she was very uncomfortable.

Participants compared their self-assessments in each mentoring domain immediately following the training vs. prior to the training [15]. For the true pre–post surveys, overall MCA skills assessment scores increased from a median (sd) of 4.3 (.63) to 5.41 (.46), *p* < 0.002, and from a median (sd) of 4.2 (0.70) to 5.27 (0.64), *p* < 0.001 for the retrospective pre–post surveys. Of specific relevance to this project, the median (sd) competency score for “Helping mentees balance work with professional life” increased from 4.42 (1.08) to 5.83 (.84), *p* = 0.002 (true pre–post) and 4.06 (1.35) to 5.22 (1.26), *p* = 0.001 (retrospective pre–post).

## Discussion

Over half of both mentors and mentees in our national sample of dyads indicated low to moderate levels of satisfaction with work–life balance, proportions similar to those found by DeCastro in a separate sample of junior faculty with K awards [2]. Despite relatively low ratings by mentors of satisfaction with their own personal/professional balance, a significant proportion of mentors and mentees were sharing personal information as part of the mentoring relationship, and such information sharing was associated with higher ratings of work–life satisfaction by mentees. These findings supported the development of a structured mentor training curriculum focused on both improving work–life integration for mentors and encouraging interactions between mentors and mentees focused on issues of work–life integration. This was accomplished by adapting content from a leadership development program into a proven-effective research mentor training curriculum. The new *Enhancing Work–life Integration* module was well received by faculty participants and resulted in both direct benefit to the faculty mentors in the form of their own successful work–life integration experiments, as well as significant improvements in their self-assessed competency in addressing work–life issues with their mentees.

While the findings in Phase 1 from a prior randomized trial of the mentor training program cannot establish a cause–effect relationship between mentors discussing work–life issues and mentee’s perceptions of work–life satisfaction, our findings indicate that a structured approach designed to help mentors guide their mentees in managing work–life conflict can significantly improve mentors’ self-reported mentoring skills in this domain [2,10,11]. Such training can encourage constructive expansion of traditional mentoring activities to address this area of increasing importance to junior faculty and research trainees and should complement institutional policies and other efforts to facilitate better work–life integration for faculty in academic medicine [1,3,8,9].

The voluntary nature of the mentor training program evaluated in Phase 3 likely self-selected for faculty who were motivated to participate in such professional development programs and more likely to perceive benefit from participating. The nearly universal positive feedback from participating faculty with a wide range of research interests and mentoring experience indicates that such a program is likely applicable to a varied faculty phenotype in any academic medical center. However, all participating faculty were from a single academic medical center and its affiliated free-standing children’s hospital, thus limiting the potential generalizability of these findings. The *Enhancing Work–life Integration* module is now available and being used in trainings nationally via NRMN and has been adopted into other mentee training programs.

The current research program did not include longitudinal follow-up of faculty who participated in the mentor training program to gain insight on how they ultimately used the training to address issues of work–life conflict with their mentees, nor could it assess the impact of this training on their mentees’ assessments of work–life satisfaction. Faculty were encouraged to utilize the *Total Leadership* materials with small groups of their mentees who are at similar career stages to facilitate peer-to-peer mentee coaching groups akin to the experience from the training program. Future research should assess the implementation of work–life integration discussions and activities in the mentoring relationship to better define how the training is best translated into mentoring practice. In addition, further research is needed to determine the impact of this mentor training curriculum on outcomes of importance to mentees, such as their perceived satisfaction with their work–life integration and the contribution made by their mentors. This would help further guide mentors to best mediate this key topic with mentees and inform institutional practices to address work–life conflicts.
